# Age-specific seroprevalence of hepatitis A in Antananarivo (Madagascar)

**DOI:** 10.1186/1471-2334-8-78

**Published:** 2008-06-06

**Authors:** Vaomalala Raharimanga, Jean-François Carod, Charles-Emile Ramarokoto, Jean-Baptiste Chrétien, Fanjasoa Rakotomanana, Antoine Talarmin, Vincent Richard

**Affiliations:** 1 Unité d'Epidémiologie, Institut Pasteur de Madagascar, BP 1274, Antananarivo 101, Madagascar; 2 Centre de biologie clinique, Institut Pasteur de Madagascar, BP 1274, Antananarivo 101, Madagascar; 3 Direction, Institut Pasteur de Madagascar, BP 1274, Antananarivo 101, Madagascar

## Abstract

**Background:**

Hepatitis A virus (HAV) is an enteric, viral, infectious disease endemic in many developing countries such as Madagascar. Infection is often subclinical or asymptomatic in children; however, symptomatic acute infections become more common with increasing age. In some developing countries, improvements in living conditions have led to changes in the epidemiological pattern of HAV infection. There are very few reports on the prevalence of HAV in Madagascar.

This study was to determine the seroprevalence of hepatitis A virus antibodies in relation to age in the city of Antananarivo, Madagascar.

**Methods:**

Serum samples collected in 2004 during a cross-sectional survey of individuals aged between two and 24 years from Antananarivo were tested for anti-HAV antibody using a commercial enzyme immunoassay kit. Subjects were investigated using a standardized social and medical history questionnaire.

**Results:**

926 subjects were enrolled including 406 males and 520 females. There were 251 children under 10 years old and 675 subjects between 10 and 24 years old. Of the 926 serum samples tested, 854 (92.2%) were positive for anti-HAV antibodies. The number of seropositive samples was similar for males and females. The overall seroprevalence was 83.7% (210/251) for children under 10 years old and 95.5% (644/675) for subjects aged between 10 and 24 years (p < 0.001).

**Conclusion:**

Despite improvements in sanitary conditions and hygiene over the last few years, the prevalence of HAV in Antananarivo is high. Only children under five years old remain susceptible to HAV infection. Immunization against HAV is not needed at the present time in the Madagascan population, but should be recommended for travellers.

## Background

Hepatitis A virus (HAV) is an epidemiologically important virus that causes acute hepatitis in humans. Most HAV infections are transmitted via the orofecal route, either by direct contact with an infected person or by ingestion of contaminated food or water.

Low socioeconomic status, high density housing and inadequate water treatment contribute to a pattern of high endemicity in developing countries in which more than 90% of the population has acquired natural immunity before the age of 10 often from an asymptomatic infection. In such countries, overt forms of hepatitis A are relatively rare with only exceptional severe cases [1,5]. While children who become infected are usually asymptomatic or develop only mild symptoms, adults infected with hepatitis A can develop fever, asthenia and jaundice. Immunity to subsequent HAV infection is lifelong.

The epidemiological pattern of hepatitis A infection is currently changing in many developing countries: improved sanitary conditions and hygiene practices have reduced the incidence of HAV infection [1]. However, the widely asymptomatic and milder forms of infection are underreported and thus the true incidence of hepatitis A is often underestimated. Thus, the epidemiological pattern of HAV in a given country is revealed primarily by its seroprevalence and only secondarily by disease incidence.

Three epidemiological patterns of endemicity (low, intermediate and high) are observed worldwide. Each pattern has a different rate of infection, prevailing age of infection, and transmission model. HAV epidemiological patterns are highly dependent on age and level of hygiene [1-3]. The distribution of HAV seroprevalence by age group may reflect current hepatitis A endemicity in countries and regions.

This study examines HAV seroprevalence in Antananarivo, the largest and most urbanized city in Madagascar. The aims of this study were to determine the age-specific seroprevalence of HAV in a young population (between two and 24 years) according to socioeconomic status and to detect any potential change in the epidemiological pattern of infection.

## Methods

### Study design and population

Antananarivo (*Commune Urbaine d'Antananarivo or CUA*) is the capital city of Madagascar. located on the central highlands. According to a report from the civic authorities (Mairie d'Antananarivo-Ville), CUA had a population of about 1.5 million in 2004. Antananarivo consists of administrative, commercial, industrial and residential areas, with patches of agricultural land that are mostly rice fields. The city is divided into six administrative districts (*Firaisana*).

This was a seroepidemiological study of HAV in individuals from Antananarivo, aged between two and 24 years, based on a descriptive cross-sectional study carried out during March and May 2004. A two-stage cluster sampling was used. In the first stage *fokontan*y (the smallest administrative unit in Madagacar) were randomly sampled. In the second stage, households in each of these *fokontany *were sampled. All family members aged between two and 24 years in the selected households were included. Informed written consent was obtained from the participants or the parents of children. The study was conducted as part of a collaboration between the Ministry of Health of Madagascar and the Pasteur Institute of Madagascar and was reviewed and approved by the National Ethical Committee.

#### Data Collection

Epidemiological data were collected from questionnaires and included general information about the target population (age, sex, educational level of the head of the household and socioeconomic characteristics including the number of siblings, type of residence, water supply, area of residence) as well as any medical history of jaundice, Hepatitis B immunization or use of traditional medicines. Blood samples were collected for HAV serology Serum samples were tested for IgG antibodies to HAV using a qualitative enzyme immunoassay kit (Monolisa^® ^anti-HAV IgM EIA, BIO-RAD, France). Results were read on a multimode plate reader and were compared with the optical densities of positive and negative controls.

#### Statistical analysis

Data were analysed using EPI Info software, Version 6 (Centers for Disease Control and Prevention, Atlanta, USA). A descriptive analysis was followed by bivariate analysis using χ^2 ^test or one-factor ANOVA for comparison of the various sub-groups with p < 0.05 defined as the minimal level of significance. Factors with p < 0.20 were subjected to a multivariate analysis with logistic regression to determine predictive variables associated with seroprevalence. Odds ratios and 95% confidence intervals were calculated and presented for these variables.

## Results

**Figure 1:**
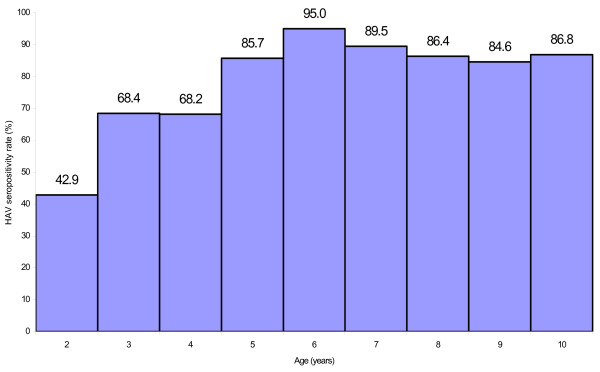
Prevalence of HAV antibodies according to age in subjects aged between two and 10 years old, Antananarivo 2004 (χ^2 ^test for trend *P *< 0.01).

The mean age of the 926 subjects was 13.9 years (95%CI [13.7–14.3]). The sex ratio was 0.78 (406 males/520 females). Of the 926 serum samples analysed for the anti-HAV (IgG) antibody, 92.2 % (854/926) were positive. Anti-HAV seroprevalence was measured for the following age groups: [2–5[, [5–10[, [10–15[, [15–20[and [20–25[. As Table 1 shows, anti-HAV seropositivity rate increased with increasing age (Univariate χ^2 ^test for the trend *P *< 0.001). Approximately 95.4% of subjects in age groups ≥ 10 years were seropositive for anti-HAV antibodies compared with children aged between two and 10 years (64.6% in [2–5[years and 88.2% in [5–10[years). In children aged between five and 10 years old, the anti-HAV seropositivity rate was more than 90% (Figure 1). Overall, there was no significant difference (p = 0.16) in the probability of being anti-HAV seropositive between females (93.1%) and males (91.1%). The seroprevalence rate was highly correlated with age, socioeconomic status and access to clean water and sanitation (Table 1).

**Table 1:** Seroprevalence of antibodies and known risk factors for hepatitis A virus (HAV) in a Madagascan population aged between two and 24 years old.

**Variable**	**Total, no.**	**(%)**	**HAV + no.**	**(%)**	**Odds ratio**	(95% CI)*	**Adjusted Odds Ratio**	(95% CI)*
**Sex**								
Male	406	(43.8)	370	(91.1)	1			
Female	520	(56.2)	484	(93.1)	1.3	(0.8 – 2.1)		
								
**Age group**								
[2–5] years	48	(5.2)	31	(64.6)	1		1	
[5–10] years	203	(21.9)	179	(88.2)	4.1	(1.9 – 8.4)	4.7	(2.1 – 10.8)
[10–15] years	248	(26.8)	233	(94.0)	8.5	(3.8 – 18.7)	11.7	(4.8 – 28.4)
[15–20] years	208	(22.5)	197	(94.7)	9.2	(4.2 – 22.9)	15.1	(5.8 – 38.9)
[20–25] years	219	(23.6)	214	(97.7)	23.5	(8.1 – 68.2)	37.7	(11.8 – 120.6)
								
**Occupation**								
Professional	110	(12.1)	93	(84.5)	1		1	
Manual Workers	806	(87.9)	751	(93.2)	2.5	(1.4 – 4.5)	2.0	(1.0 – 4.0)
								
**Area of residence**								
Firaisana I	220	(23.8)	204	(92.7)	3.1	(1.5 – 6.1)		
Firaisana II	131	(14.1)	120	(91.6)	2.6	(1.2 – 5.7)		
Firaisana III	109	(11.8)	88	(80.7)	1			
Firaisana IV	171	(18.5)	163	(95.3)	4.9	(2.1 – 11.4)		
Firaisana V	210	(22.7)	199	(94.8)	4.3	(1.9 – 9.3)		
Firaisana VI	85	(9.2)	94,1	(94.1)	3.8	(1.4 – 10.6)		
								
**Housing**								
*Cooking method*								
Electric, gas cooker	188	(20.4)	157	(83.5)	1		1	
Wood, Charcoal	732	(79.6)	692	(94.5)	3.4	(2.1 – 5.8)	2.6	(1.4 – 4.8)
*Shower unit*								
Yes	696	(75.2)	628	(90.2)	1		1	
No	230	(24.8)	226	(98.3)	6.3	(2.2 – 20.0)	5.0	(1.7 – 14.3)
*Floor*								
Tiles, Parquet	220	(23.9)	190	(86.4)	1		1	
Wood, Soil, Cement	698	(76.1)	657	(94.1)	2.6	(1.5 – 4.2)	2.0	(1.1 – 3.6)
								
**Water supply**								
Taps, Bottle	362	(39.1)	318	(87.6)	1			
Communal Fountain	503	(54.3)	480	(95.4)	2.8	(1.7 – 4.8)		
River, Well	61	(6.6)	50	(92.6)	1.5	(0.6 – 4.5)		

* CI = confidence interval; 1 = reference.

Occupation (or parents' occupation in the case of children) had a significant effect on a subject's anti-HAV seropositivity status. Prevalence was lower (85.5%) in those with professional occupations than in manual workers (93.2%). Seroprevalence also varied according to the area of residence. Subjects living in the 3^rd ^*Firaisana *had a lower prevalence (80.7%, *P *< 0.01) than those in other *Firaisana *(Table 1).

For variables related to the quality of housing (Table 1), the type of floor (wood, soil, cement versus tiles, parquet, OR = 2.6, *P *< 0.01), cooking method (wood, charcoal vs electric, gas cooker, OR = 3.4, *P *< 0.01) and shower unit (absence vs presence, OR 6.3, *P *< 0.01) were significantly associated with a higher prevalence of HAV antibodies. Drinking water supply (Table 1) was also significantly associated with HAV seroprevalence rate (*P *< 0.01) but was more reflective of the overall socioeconomic status of participants. At the time of study, 93.4% of population drank for communal water sources (standpipes or drinking fountains). Multivariate analysis confirmed the specific effects of age and economic factors: occupation, housing level (type of floor, shower unit, cooking method). The probability of being anti-HAV seropositive was significantly higher for subjects ≥ 5 years than for individuals aged [2–5[years (odds ratio 6.1, 95% CI [2.5–14.7] for [5–10[years, 14.4 [5.6, 36.8] for [10–15[years, 20.3 [7.2, 57.1] for [15–20[years, 45.4 [13.4–152.9] for [20–25[years, adjusted for other factors) (Table 1).

## Discussion

In Madagascar, available epidemiological data on HAV infection are limited. This was the first population based survey of immunity to HAV infections in urban inhabitant in Madagascar. The overall prevalence of anti-HAV in inhabitant in Antananarivo under 25 years old was 92.2%. From five years old, the majority of children (>90%) had natural immunity against HAV. These results were similar to those of other studies carried out in developing countries where the epidemiological pattern of HAV has not changed (6–8). Hepatitis A is endemic to many regions. However many recent reports indicate a global change in seroepidemiological patterns of hepatitis A infection [9,10]. In Delhi (India), seroprevalence in people younger than 35 years old was similar to that of the more developed European countries [9]. In Thailand, the prevalence of anti-HAV was 1.95% in Bangkok and 12.7% in other provinces in people younger than 25 years [10]. In Italy like in many Western European countries [11], following the improvement of the standard of living in the industrialized world, age-specific HAV seroprevalence in the general population has decreased steadily and risk of acquiring HAV infection is high for travellers to high HAV endemic areas in developing countries.

Typically, improved sanitation facilities are provided in metropolitan regions first; thus, exposure to HAV remains more common in rural regions and small cities where it principally affects the youngest inhabitants. This study in an urban setting suggested that Antananarivo, and likewise Madagascar, were hyperendemic for HAV with very high infection rates in the first years of life and most of the population acquiring antibodies to HAV before 10 years of age. HAV seroprevalence remained high in all age groups and changed with age under five years old. This finding confirmed that the greatest exposure still occurs early in life and that individuals acquire HAV immunity at a very young age. In Madagascar a previous study showed that rural regions have a high seroprevalence of HAV infection, with an estimated rate of 94.6% [12]. Only in children younger than 5 years, seroprevalence was higher in rural area (80%) than in our study in urban area (64.6%). These results showed that in Madagascar exposure to HAV was probably earlier in rural areas than in urban areas.

Regarding sociodemographic variables, our findings are consistent with several previous studies showing a clear inverse correlation between exposure to HAV and socioeconomic level [13-17]. It is well known that HAV infection is strongly correlated with poverty and inadequate sanitation. Increasing household income, education, water quality and quantity, sanitation, and hygiene lead to decreased HAV prevalence. Indeed, the prevalence of HAV infection could even be used as an index of the level of development in a given country. Prevalence of the disease varies widely, as a consequence of basic sanitation conditions.

Hepatitis A virus (HAV) is a health problem in countries where seroepidemiology shows changes from hyperendemicity to intermediate endemicity [18-21]. Then there are different patterns with pockets of low prevalence; some studies indicate a decline of anti-HAV prevalence among urban children and the possibility of an outbreak of HAV infection among this population. The infection is predicted to shift to adulthood with more severe clinical manifestation in future. Then it is important to determine the pattern of hepatitis A virus infection in each community in order to optimize vaccination strategies: high risk population for vaccination should be identified. In Madagascar, this study showed that HAV infection was highly endemic in urban areas. There are no different patterns between urban and rural areas. But similar seroprevalence studies must be conducted to detect eventually shifting of HAV endemicity level in Madagascar in order to introduce vaccination strategy.

## Conclusion

In conclusion, the precocious hepatitis A seroconversion observed in our study suggests Madagascar may be still classified among areas of high endemicity. Potential effects of improvements in hygiene and socioeconomic conditions in this metropolitan area were not detected. The inhabitants acquired a lifelong immunity as a result of natural infection during childhood. These findings do not support the implementation of mass vaccination but advocates vaccination of travellers against hepatitis A. The surveillance of the epidemiological trend of HAV infection will contribute to the definition of endemicity level in Madagascar for implementing preventive measures and for controlling the disease.

## Competing interests

The authors declare that they have no competing interests.

## Authors' contributions

VRa and VRi designed and coordinated the study. J–FC and J–BC coordinated the immunometric assay. C–ER and FR collected sociodemographic and epidemiological data. VRa and VRi analysed and discussed the associations obtained. All authors participated in data analysis. VRi drafted the manuscript. All authors read and approved the final version of the manuscript.

## Pre-publication history

The pre-publication history for this paper can be accessed here:


